# Double Myopic Choroidal Neovascular Membranes (CNVMs): A Case Report

**DOI:** 10.7759/cureus.86567

**Published:** 2025-06-22

**Authors:** Asli Perente, Aikaterini Giannoukaki, Doukas Dardabounis, Tryfon Rotsos, Georgios Labiris

**Affiliations:** 1 Ophthalmology Department, University Hospital of Alexandroupolis, Alexandroupolis, GRC

**Keywords:** anti-vegf, choroidal neovascular membrane, myopic choroidal neovascular membrane, oct angiography, pathologic myopia

## Abstract

Myopic choroidal neovascularization (CNV) is the most common cause of visual morbidity in patients with pathologic myopia (PM). Early diagnosis and management are crucial to prevent permanent loss of central vision. Optical coherence tomography (OCT) and OCT angiography (OCT-A) are the key diagnostic modalities for identifying and monitoring this vision-threatening complication. We report an unusual case of a female patient with two myopic CNVs in the same eye, clearly depicted using OCT-A and successfully managed with anti-vascular endothelial growth factor (anti-VEGF) injections. This rare instance warrants further study to draw more definite conclusions regarding this common complication and its response to treatment.

## Introduction

The incidence of myopia is steadily increasing and has become a global epidemic. Approximately 28.3% of the world’s population is affected by myopia [1], a percentage projected to rise to 50% by 2050 [2]. High myopia is defined as an axial length greater than 26.5 mm or a refractive error of ≥−6.00 diopters. When high myopia is accompanied by degenerative changes, such as myopic maculopathy and/or posterior staphyloma, the condition is referred to as pathologic myopia (PM) [3]. PM is the second leading cause of irreversible vision loss in individuals over 65 years in Taiwan [4] and the most common cause of blindness among individuals aged 40 to 49 years in China [5]. It is also the third leading cause of permanent vision loss in Western countries [6-8].

The most devastating complication of PM is the development of choroidal neovascularization (CNV), with a reported incidence between 5% and 10%. CNV refers to the abnormal growth of neovascular tissue beneath the retina and/or retinal pigment epithelium (RPE). If left untreated, CNV leads to severe vision loss and permanent chorioretinal atrophy. Early detection and timely intervention are essential. The introduction of optical coherence tomography (OCT) and OCT angiography (OCT-A) has significantly improved the ability to diagnose and follow up on CNV [9]. OCT-A is a noninvasive, depth-resolved imaging of blood flow without dye injection allowing the early detection of neovascular membranes. Several studies have investigated myopic CNV; however, to our knowledge, no previous report has described two distinct neovascular networks in the same eye of a myopic patient. Here, we describe a unique case of two distinct CNVs in the same eye of a patient with PM, a presentation not previously documented in the literature.

## Case presentation

A 64-year-old female presented to our outpatient service with a complaint of decreased vision in her left eye (OS) for several days. Her ocular history revealed high myopia with a refractive error of -6.00 D in the right eye (OD) and -8.50 D in the left eye (OS). Her medical history included hypertension and hyperlipidemia. On examination, her best-corrected visual acuity (BCVA) was 20/25 OD and 20/63 OS. Intraocular pressure (IOP) was within normal limits, and an anterior segment examination was unremarkable in both eyes.

**Figure 1:**
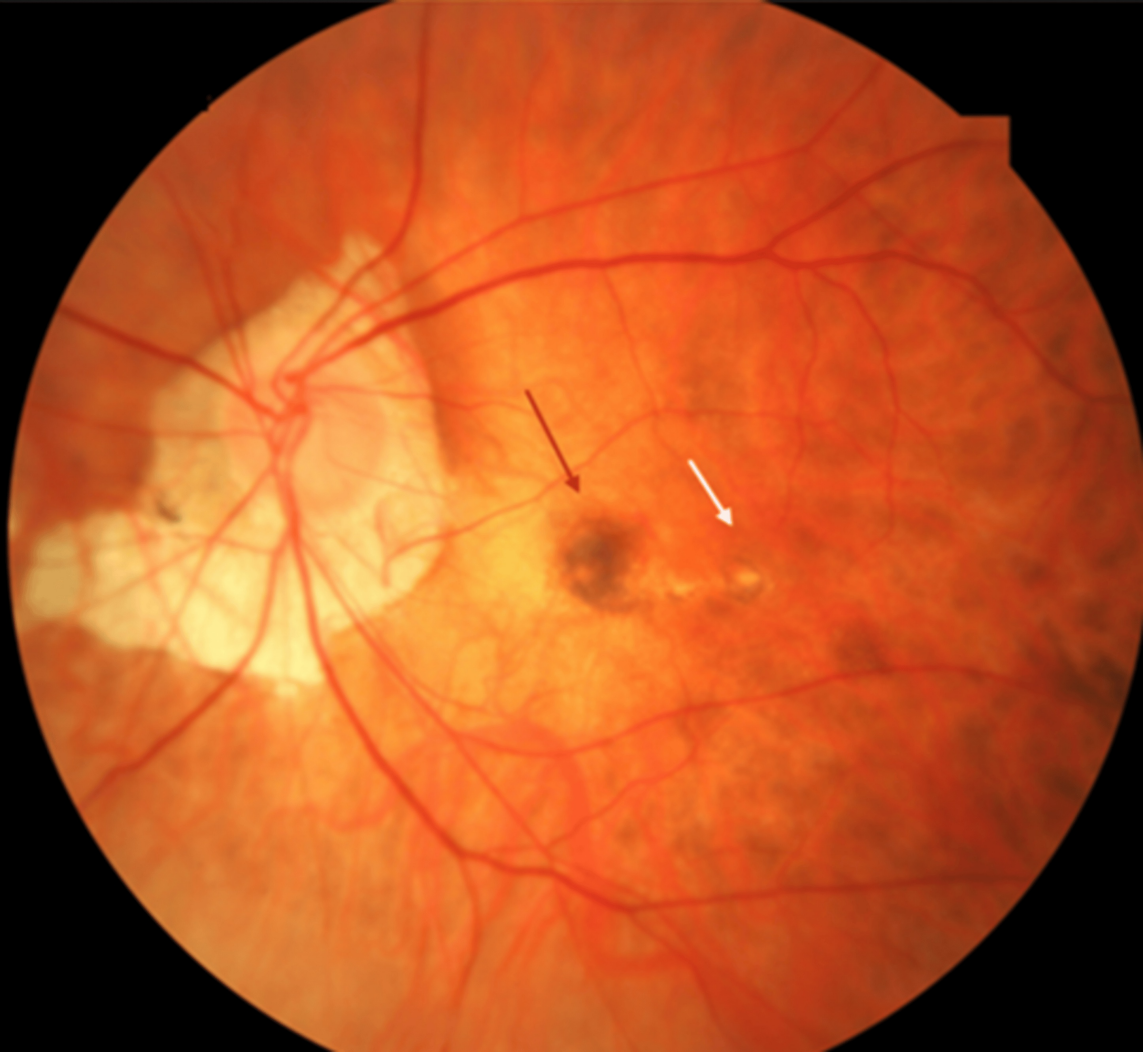
Color fundus image of the left eye (OS) showing chorioretinal atrophy and Fuchs spot (red arrow) with adjacent greyish lesion in the fovea (white arrow)

Amsler grid test was normal in OD, but she exhibited obvious metamorphopsia in OS. Fundoscopy revealed signs of degenerative myopia, including peripapillary atrophy and a tessellated fundus in both eyes. In OS, we observed chorioretinal atrophy, a Fuchs spot, and a grayish lesion at the fovea (Figure 1).

**Figure 2:**
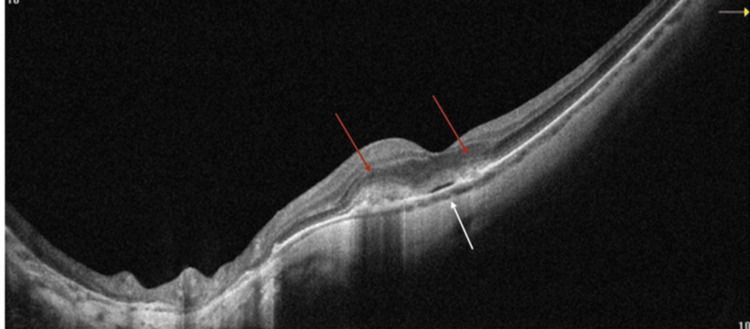
OCT of the left eye (OS) indicating two hyperreflective lesions above the RPE (red arrows) with SRF (white arrow) OCT: optical coherence tomography, RPE: retinal pigment epithelium, SRF: subretinal fluid.

**Figure 3:**
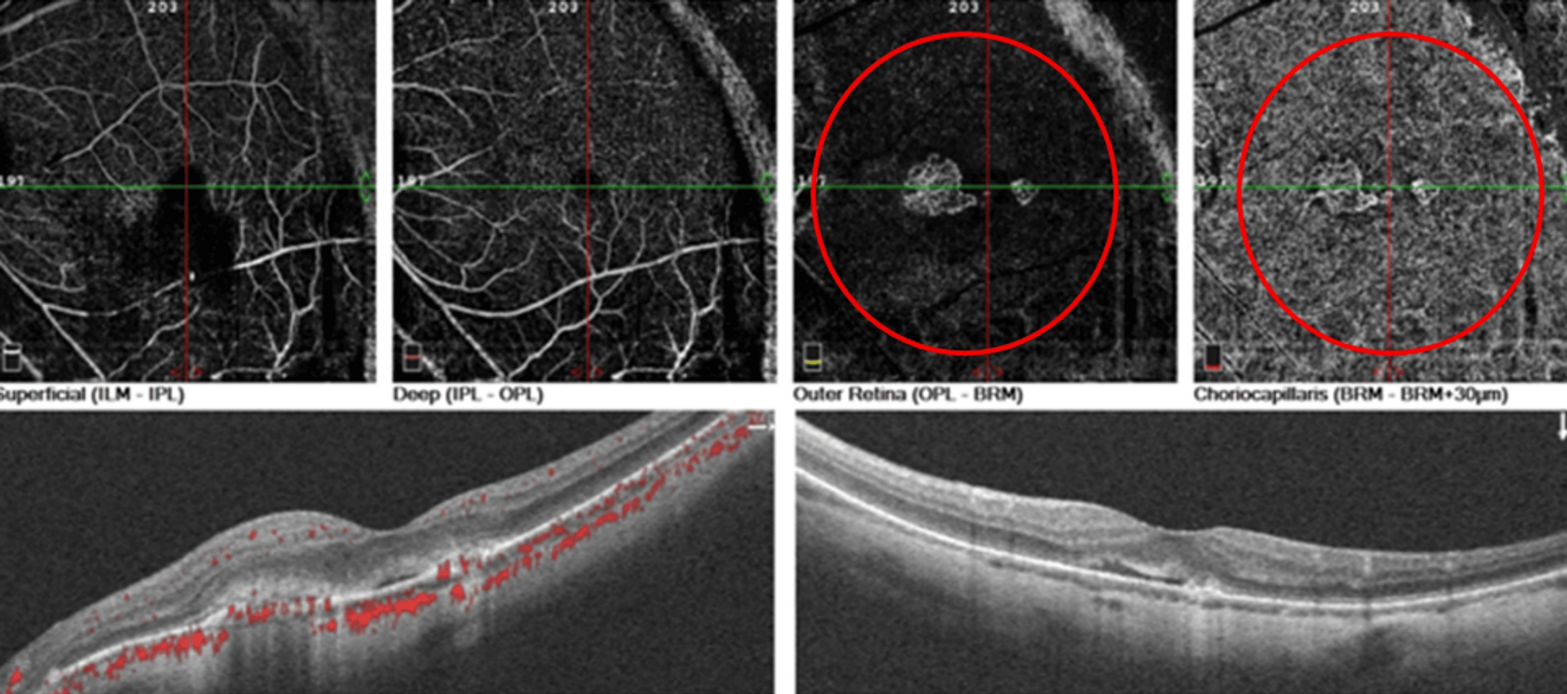
OCT-angiography of the left eye (OS) showing two separate lacy networks of neovascularization (red circles) confirming the presence of dual myopic CNVs The bottom-left image is a horizontal OCT scan, and the bottom-right image is a vertical OCT scan. OCT: optical coherence tomography, CNV: choroidal neovascularization, LPT: lesion projection thickness, ILM: internal limiting membrane, IPL: inner plexiform layer, OPL: outer plexiform layer, BRM: Bruch’s membrane.

**Figure 4:**
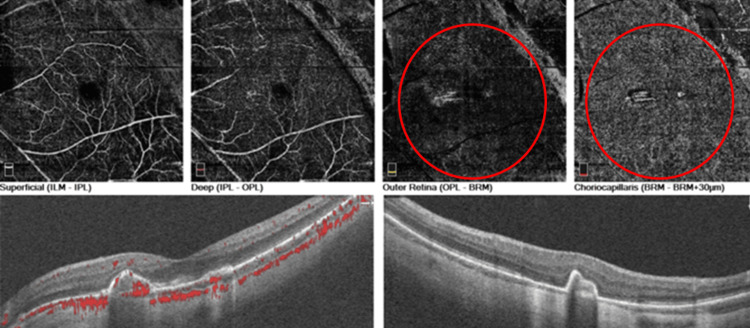
OCT-angiography of the left eye (OS) after two injections of ranibizumab with complete resolution of SRF and inactivation of CNVs (red circles) with RPE cover The bottom-left image is a horizontal OCT scan, and the bottom-right image is a vertical OCT scan. OCT: optical coherence tomography, CNV: choroidal neovascularization, RPE: retinal pigment epithelium, SRF: subretinal fluid, LPT: lesion projection thickness, ILM: internal limiting membrane, IPL: inner plexiform layer, OPL: outer plexiform layer, BRM: Bruch’s membrane.

OCT of OS showed two subretinal hyperreflective areas above the RPE with minimal subretinal fluid (Figure 2). OCT-A showed two separate lacy networks of new vessels above the RPE, suggestive of two myopic CNVs (Figure 3). We initiated treatment with intravitreal anti-vascular endothelial growth factor (anti-VEGF) injections. After two monthly injections of ranibizumab, the patient’s BCVA improved to 20/32 OS. Monthly follow-up over a six-month period showed inactive CNVs with RPE coverage on OCT and OCT-A at each visit (Figure 4).

## Discussion

Myopic CNV is the most common type of CNV in several countries, especially among young patients [10]. CNV typically progresses through three phases: active, scar, and atrophic [11]. Diagnosing the condition during the active phase and initiating prompt treatment are crucial to preventing permanent vision loss.

Traditionally, fluorescein angiography and indocyanine green angiography have been considered the gold standards for visualizing the retinal and choroidal vasculature [12]. However, OCT-A, a noninvasive imaging modality, provides valuable information on both retinal and choroidal circulation. It is reproducible and allows for assessment of flow changes in various layers of the retinal and choroidal microcirculation as well as the tracking of changes over time [13,14]. According to Querques et al. [15], OCT-A has a sensitivity of 90.48% and specificity of 93.75% for diagnosing myopic CNV, whereas fluorescein angiography has a sensitivity of 47% and specificity of 80.4% [16]. In our case, OCT-A clearly depicted two separate neovascular networks, confirming the diagnosis of two distinct myopic CNVs.

In terms of the underlying cause, the pathogenesis of myopic CNV is not fully understood. In addition to genetic predisposition, both mechanical and hemodynamic theories have been proposed. Specifically, excessive elongation of the eyeball causes mechanical stretch in multiple layers of the eye, resulting in degenerative alterations that predispose the eye to CNV formation. Several studies have reported that this mechanical stress may disrupt the balance between pro- and anti-angiogenic factors, contributing to CNV development.

According to the hemodynamics theory, perfusion changes in myopic choroidal circulation lead to hypoxia, which stimulates the increased expression of VEGF. VEGF promotes neovascularization, explaining both the pathology and the efficacy of anti-VEGF treatments for myopic CNV [17]. However, Du et al. noted that the different response to anti-VEGF treatment between myopic CNV and CNV secondary to age-related macular degeneration warrants further investigation [18].

## Conclusions

This report highlights a rare case of a myopic patient with two separate active CNVs in the same eye. Both CNVs became inactive following two anti-VEGF injections. In treating myopic CNV, clinicians generally avoid the loading dose and administer treatment only when the membrane is active. This approach is based on the typically favorable response to anti-VEGF therapy in myopic CNV as well as on the increased risk of chorioretinal atrophy associated with consecutive repeated injections. However, this case presents a particular challenge, as the presence of two distinct active CNVs introduces uncertainty regarding the natural course of the disease and the long-term treatment response. The unique nature of this case, combined with the distinct pathophysiology of myopic CNV, underscores the need for further research into this vision-threatening condition.
